# PCR Technology for Detection of Invasive Aspergillosis

**DOI:** 10.3390/jof2030023

**Published:** 2016-08-11

**Authors:** Rosemary A. Barnes, P. Lewis White

**Affiliations:** 1 Department of Medical Microbiology and Infectious Diseases, Cardiff University School of Medicine, Cardiff CF14 4XN, UK; 2 Public Health Wales Microbiology, Cardiff CF14 4 XW, UK; Lewis.white@wales.nhs.uk

**Keywords:** *Aspergillus* infection, *Aspergillus* PCR

## Abstract

The application of molecular technologies to aid diagnosis and management of infectious diseases has had a major impact and many assays are in routine use. Diagnosis of aspergillosis has lagged behind. Lack of standardization and limited commercial interest have meant that PCR was not included in consensus diagnostic criteria for invasive fungal disease. In the last ten years careful evaluation and validation by the Aspergillus European PCR initiative with the development of standardized extraction, amplification and detection protocols for various specimen types, has provided the opportunity for clinical utility to be investigated. PCR has the potential to not only exclude a diagnosis of invasive aspergillosis but in combination with antigen testing may offer an approach for the early diagnosis and treatment of invasive aspergillosis in high-risk populations, with the added benefit of detection of genetic markers associated with antifungal resistance.

## 1. Introduction

The availability of sensitive molecular methods for the diagnosis of infectious diseases is widespread, but polymerase chain reaction (PCR) for the diagnosis of fungal infection, particularly aspergillosis, is still not widely used. Difficulties in extracting nucleic acid (NA) and detecting specific NA sequences unique from other eukaryotic organisms plus difficulties in the interpretation of results has hindered the development of commercial assays. This has resulted in a plethora of in–house assays using different samples, targets, platforms and overall methodologies [1].

These ubiquitous environmental organisms are common laboratory contaminants but relatively rare causes of invasive disease occurring predominantly as opportunist infection in severely immunocompromised hosts. Nonetheless, invasive aspergillosis is a devastating disease with a high mortality [2]. Early intervention with antifungal treatment can improve outcomes [3] but the diagnostic difficulties mean few infections are proven during life. Conventional microbiological culture is insensitive and diagnosis requires histological evidence from tissues [4]. Many infections are only diagnosed at autopsy. Consequently, treatment is often used empirically in patients considered at risk with refractory fever and signs of infection [5]. This empirical usage is very costly and can have adverse effects on the patients causing organ toxicity, drug interactions and has the potential to promote antifungal drug resistance. There is no high quality evidence to show that empirical therapy improves outcomes for patients and most patients treated empirically do not have invasive fungal disease [6]. There may be a potential benefit in starting empirical therapy where diagnostic tests are not rapidly available but it should be considered a temporary measure until invasive disease can be excluded.

Several biomarkers, galactomannan (GM) and β-d-glucan are commercially available to assist in diagnosed of aspergillosis but performance varies greatly depending on the population tested and the prevalence of disease. Utility is best understood in high risk groups such as adult allogeneic stem cell transplant patients, and neutropenic patients with fewer data on paediatric and solid organ cancer patients [7].

Consensus definitions of invasive mould disease have been developed by the European Organization for Research and Treatment of Cancer/Invasive Fungal Infections Cooperative Group and the National Institute of Allergy and Infectious Diseases Mycoses Study Group (EORTC/MSG) Consensus Group [8]. They are designed to provide consistency for use in clinical drug trials and not for assessment of diagnostic accuracy.

The categories of probable and possible invasive aspergillosis are used to indicate established disease, a diagnosis reached on a basis of suggestive radiological signs with or without the detection of biomarkers (Galactomannan or β-d-glucan) or culture of *Aspergillus*. Sensitivity and specificity of biomarkers has been evaluated mainly through retrospective studies in pre-selected populations, which do not examine the performance of a test in terms of diagnostic accuracy. When applied prospectively to selected high-risk groups, such as allogeneic stem cell transplant patients, both assays show good sensitivity and a high negative predictive value [9,10]. The pan-fungal biomarker, β-d-glucan may be particularly useful for excluding disease but the relatively high number of false positive results combined with the low prevalence, limit the ability to predict disease [11]. Galactomannan testing of bronchoalveolar lavage fluid has shown good predictive value in haematological patients [12,13,14].

Sensitivity of radiological signs varies from 25% to 95% in adults and in children is even lower [15,16]. Chest X-rays are rarely helpful and computerized tomographic signs whilst suggestive of disease, are non-specific. Studies lack standardization and interpretation is subjective [17]. Validation of radiological markers as a diagnostic tool is limited and although they demonstrate the best ability to predict disease this reflects in part, the dependence of the consensus diagnostic criteria on specific radiological signs. Galactomannan or β-d-glucan are also included within the consensus definitions and in many practice guidelines although ability of these tests to alter patient management and influence treatment is difficult to determine.

PCR is not yet included in the consensus EORTC/MSG definitions, although recently *Aspergillus* PCR has been proposed for inclusion in the revision of these definitions [18]. A variety of platforms offer potential for real-time detection of nucleic acid from an array of pathogens. Most are in-house assays using a variety of protocols involving different specimen types, extraction techniques, molecular targets, amplification platforms and detection of the amplicon. This, and the lack of standardization, has hampered the acceptance of fungal NA detection.

As ubiquitous environmental organisms, fungi are common contaminants in the laboratory and fungal DNA is frequently present in reagents including molecular grade products such that stringent controls and containment are required to limit and detect contamination. However, the advantages of a rapid and highly sensitive molecular diagnostic test outweigh these problems.

## 2. Recent Advances

The European Aspergillus European PCR initiative (EAPCRI) was set up as a working group of the International Society of Human and Animal Mycology (ISHAM) in 2006. The primary aim was to develop a standard for *Aspergillus* PCR methodology and to validate this in clinical trials. The EAPCRI is now a global initiative with 86 participants in more than 60 centres across Europe and now including collaborators in the Middle East, North and South America and Australia.

Systematic studies using a series of specimen panels sent out from 2007 onwards to participating laboratories across Europe and Australia followed by meticulous analysis of results and methodologies, have resulted in published standardised methodology for whole blood, serum and plasma [19,20,21,22]. Importantly, the EAPCRI has assessed the critical stages *Aspergillus* DNA extraction and amplification [23]. Published protocols ensure optimal performance across laboratories through identification of the critical phases of the process.

Initial studies using whole blood showed that sample input volume, sample processing steps (red and white cell lysis and bead beating), fungal cell lysis through bead beating and elution volume all affected PCR performance and led to the conclusion that *Aspergillus* PCR efficiency is limited by extraction and not by PCR amplification [21,23]. Whilst whole blood testing potentially enables both intracellular, cell free DNA and cell-associated DNA to be targeted, the pre-analytical processing steps are complex and time consuming and differ considerably to those used in routine molecular diagnostic laboratories. The use of serum or plasma is technically less demanding and more suited to routine laboratories. Whilst marginally (but not statistically significantly) less sensitive than whole blood, serum targeting free DNA showed less false positivity (sample false positivity: 1.7% for serum vs. 5.7% for whole blood [24]). The use of plasma as a specimen avoids the potential loss of free DNA due to trapping during clot formation and may improve clinical performance [25]. 

Whilst published protocols for other specimens (tissues, cerebrospinal fluid, bronchoalveolar lavage (BAL) fluid) are not yet available, many of the principles already identified by the EAPCRI should apply. International efforts at methodological standardization of BAL fluid are in progress and due for completion in the next 12 months. The focus being the ability to detect both cell free DNA and cell associated DNA using a single protocol.

These standardized methodologies are all suited to automated extraction systems and PCR platforms [26] and have paved the way for commercial assays to be introduced. By combining commercial PCR assays with international NA extraction recommendations, a fully standardized molecular method should be possible. The benefits of using standardized methodologies was illustrated by a meta-analytical review where there was trend towards increased sensitivity and a significant increase in specificity for assays in line with EAPCRI recommendations compared to non-compliant methods [27].

The Invasive Aspergillosis Animal Model (IAAM) group and *Aspergillus* technology (AsTec) consortium have developed, through collaboration with the EAPCRI, a quantified a DNA calibrator that can be used to compare PCR amplification methods negating the need for a single PCR test [28] It is hoped that this calibrator can be used to develop an international standard sample so that the entire molecular process can be monitored and compared, in line with PCR tests for other infectious disease (e.g., HIV, Hepatitis C and CMV). The availability of independently developed external quality control schemes for PCR (e.g., Quality Control for Molecular Diagnostics *Aspergillus* PCR panel) will allow centres to compare performance against peers and in doing so identifying any potential short-comings in performance. The availability of calibrators, quality control schemes and guidelines on reporting real-time PCR results [29] have all helped increase the robustness of PCR.

## 3. Clinical Evaluation

Several systematic reviews of the literature have established that PCR has the analytical and clinical sensitivity and specificity to provide a useful diagnostic test for *Aspergillus* disease [27,30,31] but it is important to understand whether a test is being performed to exclude (screening) or confirm a diagnosis (diagnostic). This can be influenced by the sample type being tested and interpretive criteria used (single vs. multiple positives). When testing blood samples from 230 to 374 proven/probable cases of IA and 1386–1863 control patients with no evidence of invasive fungal disease (IFD), meta-analysis generated sensitivity and specificity values of 84%–88% and 75%–76% respectively [31].

### 3.1. Screening

Regular testing of easily obtained specimens (e.g., blood) can be incorporated into a screening strategy of high-risk patients regardless of signs and symptoms. Like other biomarkers (Galactomannan or β-d-glucan), PCR shows moderate diagnostic accuracy when used as screening tests for IA in high-risk patient groups. When testing blood, specificity of both antigen tests is higher than for PCR, while the sensitivity of PCR is higher than for or β-d-glucan and PCR [18]. This sensitivity gives a high negative predictive value (NPV) such that a negative test may allow the diagnosis to be excluded. This is the intended strategic role of a screening test. Positives show good specificity but the low prevalence of disease leads to a low positive predictive value in diagnosis of IA. It is increasingly recognized that PCR positivity can reflect exposure to *Aspergillus* and may be positive long before a disease process manifests or other biomarker positivity is detected [32]. Multiple positive results improve diagnostic utility and may be used initiate pre-emptive therapy and to trigger further diagnostic work-up [33].

When combined with other biomarkers particularly antigen-based tests, specificity increases further [34]. The combination of galactomannan and PCR has been used most studies and shows moderate diagnostic utility in IA when used in high-risk haematology patients [35].

A recent meta-analysis reviewing the combined performance of *Aspergillus* PCR and galactomannan when testing 1670 high-risk haematology patients, of which 273 were diagnosed with proven/probable IA, showed that when a single galactomannan or PCR result was considered significant the combined sensitivity was greater than that of both assays when used independently [27]. Consequently, if both assays were consistently negative, then IA could be excluded. If a patient was both PCR and galactomannan positive then specificity was 98% and the positive likelihood ratio was 43.2 and highly suggestive of disease, although this was not significantly superior to the predictive value of attaining multiple positive results by either PCR or galactomannan. Several studies have shown that screening using combination testing decreases overall antifungal drug usage, particularly empirical therapy [35,36,37], results in earlier diagnosis [24,35,36,37,38] and in one study increased aspergillus-free survival [36]. The approach can be cost-effective [39] also can be used to identify patients who are unlikely to have IFD. This improved targeting of treatment is a fundamental aspect of antifungal stewardship and not only reduces unnecessary drug usage, but can control costs and minimize risks of development of antifungal drug resistance. All studies have focused on patients with haematological malignancy and undergoing stem cell transplantation. Data in other patient groups are more limited.

### 3.2. Diagnostic

Whilst PCR can be performed on blood specimens from asymptomatic patients as a screening strategy to rule out IFD (allowing a move away from empiric therapy), it can also be performed on other specimens taken during signs of suspected infection (blood, BAL fluid, tissue), providing a diagnostic test to rule in disease and allow antifungal therapy to be targeted more appropriately. Once signs of infection develop, the pre-test probability of disease increases and the clinical utility of any test may alter. The likelihood of aspergillosis will vary from low (as in the case of non-specific fever) to moderate (in the case of specific chest symptoms and radiological changes). Bronchoalveolar lavage is a relatively invasive procedure that may be contraindicated in some patients. It is generally only performed in patients with established respiratory disease and the focus of PCR should be on confirmation of diagnosis.

Several meta-analyses of *Aspergillus* PCR when testing BAL fluid taken from 245 to 319 proven/probable cases of IA and 927–1266 control patients with no evidence of IFD generated consistent sensitivity and specificity, ranging from 76.8–79.65 to 93.7–94.5, respectively [12,40,41]. With testing specimens to confirm a diagnosis, specificity is paramount, and for PCR testing of BAL samples this is high. The positive likelihood ratios generated by meta-analyses ranged from 12.4 to 13.9, highly indicative of disease. When comparing meta-analyses of galactomannan and PCR testing of BAL fluid the specificity of PCR is significantly greater (*p* < 0.0001–0.0019) [41].

Evidence of clinical utility is growing but interpretation and understanding of test results is important for rational use of resources. In clinical practice, both screening and diagnostic strategies can be used within the same patient population depending on underlying risk of IFD, prevalence and pre-test probability of disease, specimen type, use of mould-active prophylaxis and clinical suspicion of disease [33,42]. Screening strategies are probably best applied to high-risk patients not on mould-active prophylaxis. Active prophylaxis, if used effectively can lower the prevalence of disease, such that regular screening is unlikely be cost effective [43]. However, breakthrough infections can occur and a diagnostic strategy where testing is performed on the basis of clinical suspicion of infection may be useful [44,45].

## 4. Commercial Assays

Currently, the most widely validated commercial *Aspergillus* PCR assay is the MycAssay Aspergillus with performance validated in serum, BAL and tissue [46,47,48,49,50,51,52,53,54,55]. It detects a broad range of *Aspergillus* species and clinical evaluation when testing blood showed sensitivity ranging from 43.8% to 70.0% and specificity ranging from 63.2% to 100% [46,47,53,55]. Performance is optimal when testing BAL samples with sensitivity ranging from 80% to 100% and specificity ranging from 82.4% to 100% [48,50,51,54].

PCR can be designed to identify organisms to a species level and so provide not only diagnostic information but also inform on epidemiological trends and rapidly identify species/strains. Although *A. fumigatus* is the most common cause of IA, other species can also cause disease. In addition, some *Aspergillus* species have developed resistance to azole antifungals via mutation of the target gene, *CYP*51A. The determination of azole resistance in *A. fumigatus* by “in-house” molecular methods has been successful but has been limited to the use of post-amplification processes such as sequencing. This is technically demanding, prolonging reporting times and delaying results, which could influence the choice of optimal antifungal drug therapy. The development of PCR to identify resistance using real-time technology can overcome this issue. The commercially available PathoNostics Asper Genius (PathoNostics, Maastricht, The Netherlands) is a multiplex real-time PCR assay capable of detecting *A. fumigatus*, *A. terreus* and *Aspergillus* species. The assay generated a sensitivity and specificity of 88.9% and 89.3%, respectively, when testing BAL fluid samples [56]. Additionally a second multiplex PCR reaction has the ability to detect the four most prevalent mutations inferring azole resistance (TR34, L98H, Y121F and T289A) in the *CYP*51A gene of *A. fumigatus.* The assay has also been evaluated for use on serum samples and showed good clinical performance with the ability to detect azole resistance provided the fungal burden was high enough [57].

An alternative assay with the ability to detect *Aspergillus* in combination with resistance markers is the Mycogenie *A. fumigatus* qPCR assays (Ademtech, Pessac, France), although currently, validation is lacking.

## 5. Future Developments

Polymerase-chain reaction/electrospray ionization-mass spectrometry has been successfully used for the direct detection of a broad range of pathogens in a variety of clinical samples. Data for diagnosis of fungal infection particularly IA are limited to respiratory specimens and impacts on patient management have not been demonstrated [58]. However, the broad range molecular detection of fungi by new technologies, such as the Abbott IRIDICA, potentially replacing of culture, is intriguing. The multi-centre RADICAL clinical trial compared the performance of the IRIDICA and conventional systems on testing blood, respiratory samples and tissues in the critically ill, but the cohort was limited in respect to cases of IA [59]. It will be interesting to see if a single “one size fits all” approach to nucleic acid extraction will be feasible for the detection of fungi, given the previous difficulties in standardizing extraction to optimize *Aspergillus* PCR performance. It is also necessary to see how well the fungal assay performs when testing samples that could contain multiple fungal species. This is particularly relevant for respiratory samples, where commensal organisms may be present at concentrations similar to, or greater than the pathogen of interest.

Isothermal amplification is a robust molecular method that does not need a thermocycler. This greatly reduces equipment costs and raises the possibility of the development of “point of care” or near patient tests. Panfungal, species specific and genus specific probes can be combined with a biotin-peroxidase spot detection system that can be read with the naked eye [60].

Currently the contribution of these new technologies to the diagnosis of aspergillosis is unknown.

## 6. Conclusions

*Aspergillus* PCR has come a long way in the last decade and is establishing a place in mainstream diagnostics. Cost effectiveness and clinical utility still need to be further demonstrated particularly when combined with other biomarkers within a testing strategy applied to high-risk patients. Importantly, several randomized clinical trials have been completed recently and the contribution of PCR may be clearer once results have been analyzed.

The development of new technologies should be embraced to further enhance the management of patients at risk of IA.
